# Supplementary material for: Duration, frequency, and time distortion: Which is the best predictor of problematic smartphone use in adolescents? A trace data study
Source: PLoS One. 2022 Feb 18;17(2):e0263815. doi: 10.1371/journal.pone.0263815 (PMC8856513; doi:10.1371/journal.pone.0263815)
Supplement: S1 Table — (DOCX) [file pone.0263815.s001.docx]

**Supplement 1**

**Table 1**. Bivariate Pearson’s correlations among predictor and outcome variables for a weekday.

|  | **1.** | **2.** | **3.** | **4.** | **5.** | **6.** | **7.** |
| --- | --- | --- | --- | --- | --- | --- | --- |
| 1. PSU at T1 | 1 | .575^**^ | .159 | .202 | -.139 | .001 | .478^**^ |
| 1. PSU at T2 | .575^**^ | 1 | .108 | .205 | -.208 | -.095 | .326^**^ |
| 1. Trace duration of smartphone use | .159 | .108 | 1 | .547^**^ | .511^**^ | -.032 | .069 |
| 1. Trace frequency of smartphone use | .202 | .205 | .547^**^ | 1 | .088 | .159 | .328^**^ |
| 1. Δ index | -.139 | -.208 | .511^**^ | .088 | 1 | -.184 | -.183 |
| 1. Gender | .001 | -.095 | -.032 | .159 | -.184 | 1 | -.122 |
| 1. Social desirability | .478^**^ | .326^**^ | .069 | .328^**^ | -.183 | -.122 | 1 |

*p < .05; ** p < .001
