# Supplementary material for: Duration, frequency, and time distortion: Which is the best predictor of problematic smartphone use in adolescents? A trace data study
Source: PLoS One. 2022 Feb 18;17(2):e0263815. doi: 10.1371/journal.pone.0263815 (PMC8856513; doi:10.1371/journal.pone.0263815)
Supplement: S2 Table — (DOCX) [file pone.0263815.s002.docx]

**Table 2**. Bivariate Pearson’s correlations among predictor and outcome variables for a weekend day.

|  | **1.** | **2.** | **3.** | **4.** | **5.** | **6.** | **7.** |
| --- | --- | --- | --- | --- | --- | --- | --- |
| 1. PSU at T1 | 1 | .575^**^ | .020 | .091 | -.243^*^ | .001 | .478^**^ |
| 1. PSU at T2 | .575^**^ | 1 | -.013 | .023 | -.278^*^ | -.095 | .326^**^ |
| 1. Trace duration of smartphone use | .020 | -.013 | 1 | .659^**^ | .549^**^ | -.078 | -.001 |
| 1. Trace frequency of smartphone use | .091 | .023 | .659^**^ | 1 | .138 | .048 | .211 |
| 1. Δ index | -.243^*^ | -.278^*^ | .549^**^ | .138 | 1 | -.193 | -.190 |
| 1. Gender | .001 | -.095 | -.078 | .048 | -.193 | 1 | -.122 |
| 1. Social desirability | .478^**^ | .326^**^ | -.001 | .211 | -.190 | -.122 | 1 |

*p < .05; ** p < .001
