# Supplementary material for: Duration, frequency, and time distortion: Which is the best predictor of problematic smartphone use in adolescents? A trace data study
Source: PLoS One. 2022 Feb 18;17(2):e0263815. doi: 10.1371/journal.pone.0263815 (PMC8856513; doi:10.1371/journal.pone.0263815)
Supplement: S3 Table — (DOCX) [file pone.0263815.s003.docx]

**Table 3.** Spearman’s rank correlations between items of the SAS-SV at T1, traced duration and frequency of smartphone use, and difference index.

| **Items of the SAS-SV** | | | **Descriptives** | | **Spearman’s rank correlations** | | |
| --- | --- | --- | --- | --- | --- | --- | --- |
|  | **Original** | **Italian translation** | **Mean** | **Standard deviation** | **Traced duration** | **Traced frequency** | **Difference index** |
|  | Missing planned work due to smartphone use. | Non faccio i compiti in tempo a causa dell'utilizzo dello smartphone. | 1.67 | 1.00 | -0.009 | 0.013 | -0.083 |
|  | Having a hard time concentrating in class, while doing assignments. or while working due to smartphone use. | Ho difficoltà a concentrarmi in classe o a casa, quando faccio degli esercizi o mentre studio a causa dell'utilizzo dello smartphone. | 1.72 | 1.10 | -0.027 | -0.001 | -0.145 |
|  | Feeling pain in the wrists or at the back of the neck while using a smartphone. | Provo dolore ai polsi e dietro il collo mentre uso lo smartphone. | 1.59 | 1.07 | 0.140 | **.297**** | 0.057 |
|  | Won’t be able to stand not having a smartphone. | Non sarei in grado di sopportare il fatto di non avere uno smartphone. | 2.05 | 1.45 | -0.046 | 0.103 | -0.086 |
|  | Feeling impatient and fretful when I am not holding my smartphone. | Mi sento impaziente ed irritabile quando non ho in mano il mio smartphone. | 1.67 | 1.22 | 0.031 | **.260*** | -0.201 |
|  | Having my smartphone in my mind even when I am not using it. | Ho sempre in mente il mio smartphone, persino quando non lo sto utilizzando. | 1.54 | 0.95 | -0.003 | 0.130 | -0.214 |
|  | I will never give up using my smartphone even when my daily life is already greatly affected by it. | Non smetterò mai di usare il mio smartphone, nemmeno se questo mi causasse dei problemi nella mia vita quotidiana. | 1.57 | 1.15 | 0.090 | 0.054 | -0.138 |
|  | Constantly checking my smartphone so as not to miss conversations between other people on Twitter or Facebook. | Controllo costantemente il mio smartphone per non perdere le conversazioni tra i miei amici su WhatsApp o altri social network. | 2.34 | 1.44 | 0.172 | 0.105 | -0.105 |
|  | Using my smartphone longer than I had intended. | Uso il mio smartphone più a lungo di quanto previsto. | 2.27 | 1.41 | 0.093 | **.322***** | -0.145 |
|  | The people around me tell me that I use my smartphone too much. | Le persone attorno a me dicono che uso troppo il mio smartphone. | 2.08 | 1.32 | 0.214 | 0.201 | -0.035 |

*p < .05; ** p < .001; ***p<.005. Applying Bonferroni’s correction p-value should be <.005 to be statistically significant.
