# Supplementary material for: Duration, frequency, and time distortion: Which is the best predictor of problematic smartphone use in adolescents? A trace data study
Source: PLoS One. 2022 Feb 18;17(2):e0263815. doi: 10.1371/journal.pone.0263815 (PMC8856513; doi:10.1371/journal.pone.0263815)
Supplement: S5 Table — (DOCX) [file pone.0263815.s005.docx]

**Table 5**. Regression results for PSU at T1 and T2 including smartphone use on a weekday.

|  | **Outcomes** | | | |
| --- | --- | --- | --- | --- |
|  | **Problematic smartphone use at T1** | | **Problematic smartphone use at T2** | |
| **Predictor variables** | **B (S.E.)** | **β** | **B (S.E.)** | **β** |
| 1.Gender | .040 (.086) | .640 | -.104 (.081) | -.123 |
| 2.Social desirability | **-.262 (.066)** | **-.436**** | .025 (.068) | .039 |
| 3.Trace duration of smartphone use | **.102 (.053)** | **.269†** | .039 (.053) | .103 |
| 4.Trace frequency of smartphone use | -.047 (.077) | -.080 | .054 (.074) | .089 |
| 5. Δ index | -.042 (.028) | -.187 | **-.055 (.028)** | **-.233†** |
| 6.PSU at T1 |  |  | **.540 (.109)** | **.508**** |
| Intercept | .059 (.281) |  |  |  |
| Adjusted-R^2^ | .211 | | .398 | |
| F | 5.379 | | 7.949 | |
| p-value | < .001 | | < .001 | |

Legend: Δ index represents traced duration minus self-report duration; †p<.1; *p<.05; **p<.01
