# Supplementary material for: Duration, frequency, and time distortion: Which is the best predictor of problematic smartphone use in adolescents? A trace data study
Source: PLoS One. 2022 Feb 18;17(2):e0263815. doi: 10.1371/journal.pone.0263815 (PMC8856513; doi:10.1371/journal.pone.0263815)
Supplement: S6 Table — (DOCX) [file pone.0263815.s006.docx]

**Table 6** Regression results for PSU at T1 and T2 including smartphone use on a weekend day.

|  | **Outcomes** | | | |
| --- | --- | --- | --- | --- |
|  | **Problematic smartphone use at T1** | | **Problematic smartphone use at T2** | |
| **Predictor variables** | **B (S.E.)** | **β** | **B (S.E.)** | **β** |
| 1.Gender | -.013 (.084) | -.016 | -.092 (.085) | -.110 |
| 2.Social desirability | **-.258 (.062)** | **-.436**** | .043 (.068) | .070 |
| 3.Trace duration of smartphone use | **.120 (.058)** | **.337*** | .060 (.060) | .184 |
| 4.Trace frequency of smartphone use | -.082 (.069) | -.171 | -.033 (.069) | -.067 |
| 5. Δ index | **-.079 (.027)** | **-.375*** | **-.065 (.028)** | **-.302*** |
| 6.PSU at T1 |  |  | **.477 (.118)** | **.447***** |
| Intercept | .195 (.265) |  | .379 (.272) |  |
| Adjusted-R^2^ | .255 | | .316 | |
| F | 6.395 | | 6.785 | |
| p-value | < .001 | | < .001 | |

Legend: Δ index represents traced duration minus self-report duration; †p<.1; *p<.05; **p<.01
